# Factors associated with the humoral response after three doses of COVID-19 vaccination in kidney transplant recipients

**DOI:** 10.3389/fimmu.2023.1099079

**Published:** 2023-02-16

**Authors:** Ángel Bulnes-Ramos, María Mar Pozo-Balado, Israel Olivas-Martínez, Vanesa Garrido-Rodríguez, Gabriel Bernal-Blanco, Alejandro Suárez-Benjumea, Ana Isabel Álvarez-Ríos, Carmen Lozano, Carmen González-Corvillo, Marta Suñer-Poblet, Francisco Manuel González-Roncero, Berta Sánchez, Isabel Maldonado-Calzado, José Manuel Lara-Ruiz, María Francisca Gonzalez-Escribano, Yolanda María Pacheco

**Affiliations:** ^1^ Immunology Service, Institute of Biomedicine of Seville (IBiS), Virgen del Rocío University Hospital (HUVR)/CSIC/University of Seville, Seville, Spain; ^2^ Nephrology Service, University Hospital Virgen del Rocío, Seville, Spain; ^3^ Biochemistry Service, University Hospital Virgen del Rocío, Seville, Spain; ^4^ Microbiology Service, University Hospital Virgen del Rocío, Seville, Spain

**Keywords:** COVID-19, kidney transplant, mRNA vaccine, relative telomere length, thymic function, thymosin-α1, sj/β-TREC ratio

## Abstract

**Introduction:**

Kidney transplant recipients showed a weak humoral response to the mRNA COVID-19 vaccine despite receiving three cumulative doses of the vaccine. New approaches are still needed to raise protective immunity conferred by the vaccine administration within this group of high-risk patients.

**Methods:**

To analyze the humoral response and identify any predictive factors within these patients, we designed a prospective monocentric longitudinal study of Kidney transplant recipients (KTR) who received three doses of mRNA-1273 COVID-19 vaccine. Specific antibody levels were measured by chemiluminescence. Parameters related to clinical status such as kidney function, immunosuppressive therapy, inflammatory status and thymic function were analyzed as potential predictors of the humoral response.

**Results:**

Seventy-four KTR and sixteen healthy controls were included. One month after the administration of the third dose of the COVID-19 vaccine, 64.8% of KTR showed a positive humoral response. As predictive factors of seroconversion and specific antibody titer, we found that immunosuppressive therapy, worse kidney function, higher inflammatory status and age were related to a lower response in KTR while immune cell counts, thymosin-a1 plasma concentration and thymic output were related to a higher humoral response. Furthermore, baseline thymosin-a1 concentration was independently associated with the seroconversion after three vaccine doses.

**Discussion:**

In addition to the immunosuppression therapy, condition of kidney function and age before vaccination, specific immune factors could also be relevant in light of optimization of the COVID-19 vaccination protocol in KTR. Therefore, thymosin-a1, an immunomodulatory hormone, deserves further research as a potential adjuvant for the next vaccine boosters.

## 1 Introduction

COVID-19 vaccination has been demonstrated as the best tool to control the SARS-CoV-2 epidemic and it is being administered worldwide since December 2020. Unfortunately, large series of patients including kidney transplant recipients (KTR) who have already received two doses of mRNA vaccines showed poor seroconversion rates in comparison to the general population related to their immunosuppressive treatment. Different studies have shown the seroprotection rates are under 20% after the first dose, and less than 50% after the second one, while in the general population, these values are close to 100% (1–3). Although an improvement in the humoral response has been observed after a third (booster) dose, the seroconversion rate varies from 55 to 67% which is still lower than the observed in the general population (4–6).

The distribution of immune cell populations impacts the outcome of immune responses to different viral infections and vaccination settings such as the CD4/CD8 T-cell ratio in HIV-infected subjects (7), or the total CD4+ and CD8+ T-cells producing IFN-γ or TNF-α in tuberculosis and herpes zoster vaccination (8, 9). In addition, delayed reconstituted T and B cells have been related to a higher risk of viral infection after stem cell transplantation (10). In this sense, the thymus plays a main role in the generation and maturation of T-cells, and in mediating innate and adaptive immunological responses both, by the thymic output of T-cells and by the secretion of several hormones, as thymosin-α1 (Tα1), with peripheral immunomodulatory properties (11, 12). Moreover, the thymic output impacts the homeostatic proliferation of peripheral T-cells and, hence, their relative telomere length. In addition, Tα1 plasma levels and T-cell peripheral proliferation have been related to a better immune restoration in immunodeficient patients and a better recovery in SARS-CoV-2 infected patients and other immunological contexts (13–15). We hypothesized that these thymic-related parameters (thymic output, hormone secretion and immune cells relative telomere length) might be associated with the lower response to COVID-19 vaccination in immunosuppressed patients, such as kidney transplant recipients. As far as we know, the potential role of such thymic-related parameters has not been yet explored in this setting, neither in the general population. We present herein data from six months’ follow-up in a cohort of kidney transplant recipients receiving three doses of mRNA COVID-19 vaccine, including longitudinal data of humoral response and the analysis of potential predictive factors for both clinical and immunological, including distribution of main immune subsets and thymic function-related parameters.

## 2 Materials and methods

### 2.1 Study design and participants

**Figure 1:**
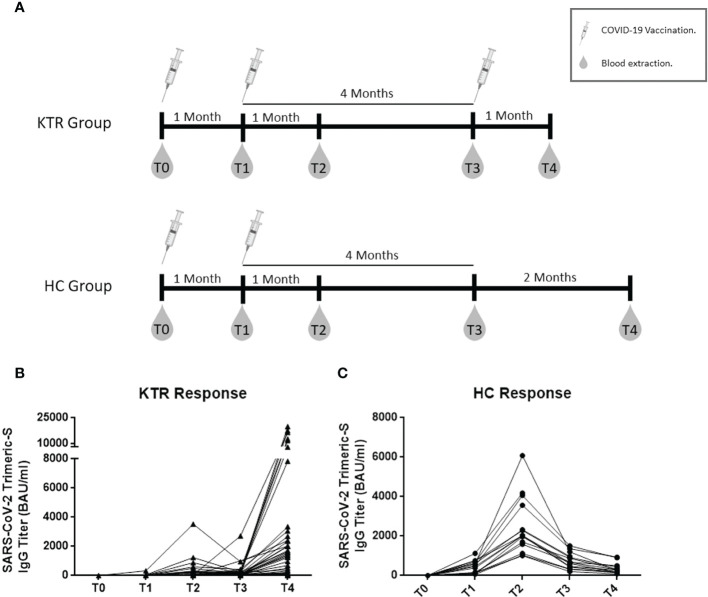
Follow-up of humoral response to COVID-19 vaccine, including design scheme of study design. Schematic study design and follow-up **(A)**, longitudinal follow-up of specific antibody IgG titers in 54 Kidney transplant recipients receiving three doses of vaccine **(B)** and in Healthy controls receiving two doses of vaccine **(C)**. KTR, kidney transplant recipients; HC, healthy controls; T0, baseline; T1, one month after the first dose and second dose administration; T2, one month after the second dose; T3, four months after the second dose and third dose administration in KTR group; T4, one month after the third dose. All comparison between time-points were statistically significant by Friedman test (*p*<0.005).

We designed a prospective monocentric longitudinal study of kidney transplant recipients (KTR) receiving three doses of mRNA-1273 COVID-19 vaccine (100 μg per dose) and healthy controls receiving two doses of BNT162b2 vaccine (30 μg per dose). KTR from the Virgen del Rocio University Hospital (Seville, Spain), were recruited if they received kidney transplant more than one month before the beginning of the study, and were older than 18 years old and signed informed consent to participate. In our region, KTR received the first dose of vaccine in April 2021. Blood samples were collected up to one month before the first dose (T0), 3-4 weeks after the first dose (T1) (i.e. just before the administration of the second dose), one month after the second dose (T2), four months after the second dose (T3) and one month after receiving the third dose (T4) (**Figure 1A**). The procedures outlined in this study were approved by the local Ethic Committee for Clinical Research (Acta number: 02/2021) and were performed according to the Helsinki Declaration of the World Medical Association.

### 2.2 Specific IgG production anti-trimeric SARS-CoV-2 spike protein

IgG antibodies against the trimeric SARS-CoV-2 Spike protein were quantified in serum samples by chemiluminescence assay (LIAISON^®^ SARS-CoV-2 TrimericS IgG, Diasorin S.p.A, Saluggia, Italy) and run on a DiaSorin LIAISON XL platform (DiaSorin, Stillwater, USA). According to the manufacturer’s data, the sensitivity and specificity of this test were 98.7% and 99.5%, showing a good correlation with microneutralization test (PPA: 100%, NPA: 96.9%). Antibody concentration, expressed as BAU/mL, was automatically calculated by the analyzer from AU/mL by the following conversion formula: AU/mLx2.6=BAU/mL. A positive result was considered as ≥33.8 BAU/mL. The levels of IgG antibodies against the trimeric SARS-CoV-2 Spike protein were considered as a continuous variable, corresponding to the magnitude of the humoral response (titer values), but they were also transformed into a dichotomous variable, corresponding to the ability to response or seroconversion, defined as an antibody titter higher or equal to 33.8 BAU/mL (the established threshold for the used assay).

### 2.3 Immune cell populations

Cell counts and percentages of lymphocytes, monocytes, neutrophils, basophils, eosinophils and platelets were measured from fresh blood samples with an Epics XLMCL flow cytometer (Beckman-Coulter, Brea, California) by standard procedures at the Immunology Service of our Hospital.

### 2.4 Soluble biomarkers

Plasma and serum samples were collected and stored at -80°C until used. Biochemical and inflammation-related biomarkers were determined by standard procedures at Biochemistry Service of the Virgen del Rocío University Hospital, at T0, in serum or plasma samples when proceeded. Quantification of the homocysteine levels were determined by photometry, whereas high sensitivity C-reactive protein (hsCRP) and β2-microglobulin levels were determined by an immunoturbidimetric assay in Cobas 701 system (Roche Diagnostics, Mannheim, Germany). eGFR was calculated by CKD-EPI index. Tα1 was determined in baseline plasma samples using Human Thymosin Alpha 1 (Tα1) Elisa kit (competitive ELISA, MyBiosurce^®^).

### 2.5 Relative telomere length

Peripheral blood mononuclear cells (PBMC) were isolated from fresh blood using density gradient centrifugation. Then, PBMC were cryopreserved until DNA was extracted by using Omega BIO-TEK, E.Z.N.A blood DNA Mini Kit. The ratio between number of copies of the telomere sequence and the single copy gen Beta-globin was determined. Copy number quantifications were performed by qPCR following a standard protocol (16). For each reaction, 60 ng of DNA were used. Primers sequences (5´-3´) were: Telomere Forward (GGTTTTTGAGGGTGAGGGTGAGGGTGAGGGTGAGGGT) and Reverse (TCCCGACTATCCCTATCCCTATCCCTATCCCTATCCCTA); Human Beta-Globin Forward (ACACAACTGTGTTCACTAGG) and Reverse (CAACTTCATCCACGTTCACC). The fluorescent reading for the copy number determination was performed in a Light-cycler 480 (Roche).

### 2.6 Thymic output quantification

Thymic output was calculated as the sj/β-TRECs ratio by Droplet digital PCR (ddPCR), in a single reaction, optimized from (17), and according to the manufacturer’s recommendations, in a QX200 system (BIORAD). The primers and probes design were optimized from (18). Each ddPCR reaction containing 150 ng of DNA from PBMC, 1x ddPCR Supermix no UTP for Probes (BIORAD), 250 nM of FAM labelled Beta Probe, 250 nM HEX labelled Delta Probe, 1 µM of 6 different Beta Forward primers, 1 µM of Beta reverse primer, 1 µM of Delta forward primer and 1 µM of Delta reverse Primers. The final volume of reaction was 20 µl. The results were analyzed by Quantasoft 1.7.1 Software

### 2.7 Statistical analysis

Statistical comparisons between groups were performed using non-parametric Mann-Whitney U-test for continuous variables or the chi-squared test for the categorical variables. For multiple longitudinal comparisons, the Friedman test applied. Correlations were explored by the non-parametric Spearman´s ρ coefficient. A p-value <0.05 was considered statistically significant. Multivariable linear or logistic regression were performed to determine independent factors affecting to antibody titers or seroconversion rates after vaccination, respectively. Those variables statistically significant in the previous bivariate analysis and those clinically or biologically relevant were included. Statistical analysis were performed using IBM SPSS v21.0 and graphics were generated with GraphPad Prism 8.0 (GraphPad Software, Inc., San Diego, CA).

## 3 Results

### 3.1 Characteristics of study subjects

Eighty-seven kidney transplant recipients (KTR) were initially recruited before receiving the first dose of the COVID-19 vaccine, but only 74 KTR were included for analysis and 54 of them remained at the last time-point (see **Supplementary Figure 1**). Demographical parameters, immunosuppression therapy, baseline kidney function and comorbidities of KTR before vaccination are detailed in **Supplementary Table 1**. Summing up, this cohort was composed of 31% females, median 59 years-old and the median period post-transplantation was 59 [14-129] months. Most of the KTR patients received corticosteroids (90.5%) (in that case, a fixed dose of 5 mg/day), tacrolimus (94.6%) and mycophenolate-mofetil (79.7%) as immunosuppressive therapy, and 10% received thymoglobulin induction. A reference group of 16 healthy controls (HC) was included (75% females, median 39 [30-45] years-old). No severe symptoms due to COVID-19 vaccine doses were reported.

### 3.2 Poor humoral response to the COVID-19 vaccine in KTR

Seroconversion rates (defined as ≥33.8 SARS-CoV-2 Trimeric-S IgG BAU/mL) were lower in KTR than HC group at T1 (12% vs. 100%, *p*<0.001) and T2 (44% vs. 100%, *p*<0.001) (**Table 1**). The KTR group achieved lower humoral titers than the HC group after the doses of vaccine (*p*<0.001 for all comparisons) (**Table 1**; **Figure 1C**).

**Table 1:** Humoral response to COVID-19 vaccination in KTR and HC group.

Humoral Response	KTR *	HC**	*p*-value
T1 seroconversion rate, n (%)	8 (12.1)	16 (100)	**<0.001**
T2 seroconversion rate, n (%)	27 (44.3)	15 (100)	**<0.001**
T3 seroconversion rate, n (%)	29 (47.5)	15 (100)	**<0.001**
T4 seroconversion rate, n (%)	35 (64.8)	15 (100)	NR***
T1 SARS-CoV-2 Trimeric-S IgG levels- BAU/mL	61 [37-109]	482 [129-711]	**<0.001**
T2 SARS-CoV-2 Trimeric-S IgG levels- BAU/mL	200 [44-573]	2020 [1580-3560]	**<0.001**
T3 SARS-CoV-2 Trimeric-S IgG levels- BAU/mL	140 [65-275]	683 [431-1020]	**<0.001**
T4 SARS-CoV-2 Trimeric-S IgG levels- BAU/mL	1576 [313-3060]	274 [176-458]	NR***

Categorical variables are expressed as n (%), and continuous variables as median [IQR]. Comparisons were tested by using Mann-Whitney U-test or χ^2^ test when proceeded. *p*-values <0.05 were considered statistically significant and shown in bold. KTR, kidney transplant recipients; HC, healthy controls; T1 one month after the first dose and second dose administration; T2, one month after the second dose; T3, four months after the second dose and third dose administration in KTR group; T4, one month after the third dose. IQR, inter quartile range. *Number of KTR included on each analysis during the follow-up: T1 (n=74), T2 (n=61), T3 (n=61) and T4 (n=54). **One HC lost the follow-up after T1. ***NR, Not relevant because HC group did not receive the third dose of COVID-19 vaccine during the period of time of this study.

Fifty-four patients remained at the follow-up after the administration of the third dose of mRNA vaccine for the analysis of the humoral response to the booster dose in those patients (**Supplementary Figure 1**). In these 54 KTR, we observed a significant increase in their antibody response, showing improved seroconversion rates (65% *vs.* 44%, *p*=0.002) and higher antibody levels than before the booster dose (1576 [313-3060] *vs.* 152 [112-279] BAU/mL, *p*<0.001). **Figure 1B** shows the full longitudinal analysis of the humoral response restricted to those 54 KTR.

### 3.3 A higher baseline thymic function improved the humoral response to COVID-19 vaccine in KTR

**Figure 2:**
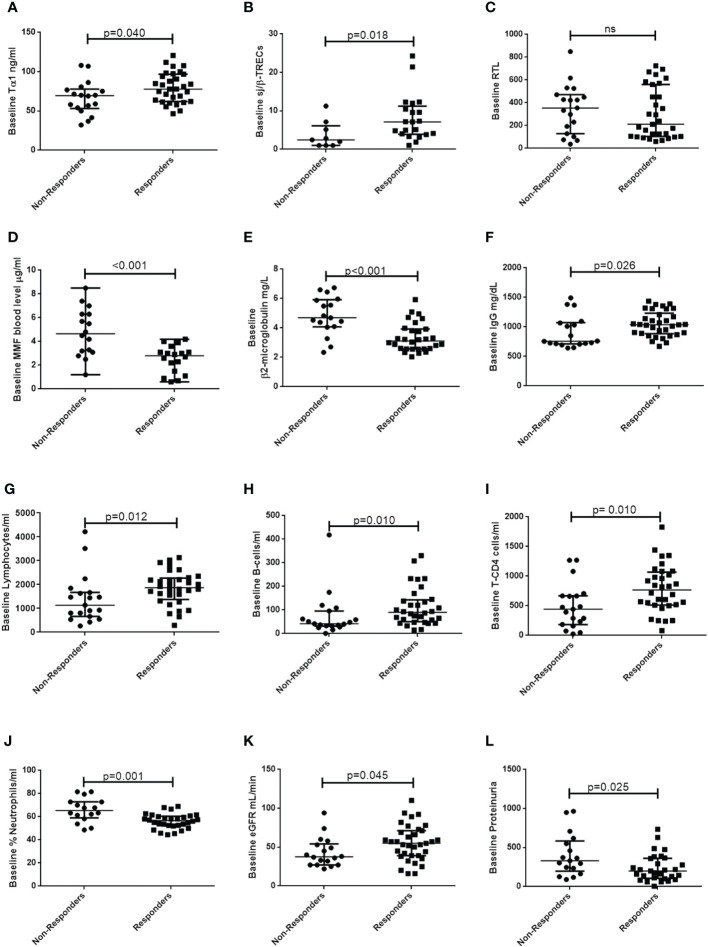
Baseline thymic function-related parameters, therapy-related factors, inflammatory-related markers, immune subsets and renal function-related factors in responders and non-responders KTR to the third dose of vaccine. Plots show comparisons of baseline levels of **(A)** Thymosin- α1 (Tα1), **(B)** sj/β TRECs ratio, **(C)** Relative Telomere length (RTL), **(D)** mycophenolate-mofetil (MMF) blood level, **(E)** β2-microglobulin, **(F)** IgG, **(G)** lymphocytes, **(H)** B-cells, **(I)** T-CD4, **(J)** Neutrophils, **(K)** eGFR and **(L)** proteinuria between responders and non-responders KTR to the third dose of vaccine. Groups were compared using Mann Whitney-*U* test, considering statistically significant a *p*-value <0.05. eGFR, estimated glomerular filtration rate, calculated by CKD-EPI index.

Seroconverted KTR patients after the third dose of vaccine showed higher baseline levels of Tα1 than non-responders (77.9 [62.1-96.8] vs. 69.5 [53.0-77.8], *p*=0.04), as well as higher baseline thymic output, measured as the sj/β-TRECs ratio (6.1 [3.7-10.8] vs. 2.2 [1.0-5.6], *p*=0.018) (**Table 2**; **Figures 2A, B**). In addition, sj/β-TRECs ratio correlated to a higher anti-trimeric SARS-CoV-2 S protein specific IgG production after vaccination (r=0.399, *p*=0.017). However, the baseline for the telomere relative length in circulating mononuclear cells did not associate with the seroconversion rate (**Figure 2C**) nor with antibody titers.

**Table 2:** Analysis of soluble markers in KTR depending of their humoral response to COVID-19 vaccination.

	T2 Response			T4 Response		
	Yes (n=27)	No (n=34)	*p*-value	yes (n=35)	no (n=19)	*p*-value
Baseline Demographical Parameters						
Female, n (%)	9 (33)	11 (32)	0.935	9 (25.7)	7 (36.8)	0.392
Age, years	53 [40-62]	62 [50-68]	**0.020**	59 [50-65]	63 [58-70]	*0.068*
Inflammation markers						
Ferritin, ng/mL	79 [45-143]	136 [74-276]	*0.072*	106 [50-179]	140 [82-280]	0.157
β2-microglobuline, mg/L	3.2 [2.5-5.4]	4.0 [3.2-5.9]	*0.057*	3.1[2.5-3.9]	4.6 [4.0-5.9]	**<0.001**
CRP, mg/L	1.1 [0.4-3.3]	1.5 [0.7-3.9]	0.405	1.1 [0.5-3.2]	1.0 [0.5-2.6]	0.465
Homocysteine, mg/L	2.6 [2.3-3.4]	3.3 [2.6-4.1]	*0.076*	2.6 [2.3-4.0]	3.4 [2.8-3.8]	0.211
Neutrophils, cells/µL	3.5 [2.7-4.4]	4.6 [3.4-5.8]	**0.035**	3.9 [3.1-5.2]	4.7 [3.1-5.6]	0.489
Neutrophils, %	55.7 [50.0-59.3]	61.4 [52.4-68.9]	**0.035**	56.6 [52.3-60.3]	65.4 [59.0-72.8]	**0.001**
Thymic Function Parameters						
Tα1, ng/mL	76.9 [61.1-88.8]	70.8 [61.0-92.5]	0.938	77.9 [62.1-96.8]	69.5 [53.0-77.8]	**0.040**
sj/β-TRECs Ratio	5.0 [3.2-11.5]	7.1 [2.0-12.2]	0.909	6.1 [3.7-10.8]	2.2 [1.0-5.6]	**0.018**
Relative Telomere Length	354 [162-572]	177 [103-489]	0.145	211 [104-560]	354 [128-471]	0.603
Baseline Kidney Function Parameters						
Creatinine, mg/dL	1.3 [1.0-1.9]	1.4 [1.1-1.9]	0.265	1.3 [1.1-1.6]	1.7 [1.2-2.2]	*0.082*
eGFR, mL/min	55.0 [39.0-77.2]	47.0 [29.5-63.0]	0.109	55.0 [39.0-71.0]	37.5 [27.0-54.2]	**0.045**
Proteinuria, mg/dL	265 [150-483]	217[135-437]	0.810	199 [106-362]	330 [198-584]	**0.025**
Immune cells populations						
Lymphocytes, cells/µL	1716 [1004-2193]	1731 [1405-2006]	0.936	1867 [1377-2269]	1134 [663-1668]	**0.012**
B-cells, cells/µL	107 [60-157]	52.0 [36.2-144.0]	*0.084*	90.0 [50.5-143.2]	42 [32-96]	**0.010**
T-CD3 cells, cell/µL	1300 [136-1895]	1439 [1157-1805]	0.847	1512 [1028-1907]	959 [431-1449]	**0.025**
T-CD4 cells, cells/µL	688 [348-1104]	666 [481-860]	0.445	767 [513-1068]	443 [184-667]	**0.010**
T-CD8 cells, cells/µL	558 [297-738]	693 [389-936]	0.274	610 [434.2-959.2]	415 [214-726]	0.131
NK cells, cells/µL	123 [50-256]	127 [56-276]	**0.041**	147 [79-289]	130 [80-211]	0.345

Categorical variables are expressed as n (%), and continuous variables as median [IQR]. Comparisons were tested by using Mann-Whitney U-test or χ^2^ test when proceeded. *p*-values <0.05 were considered statistically significant and shown in bold, whereas *p*-values between 0.1 and 0.05 were shown in italics. T2: one month after second dose of COVID-19 vaccination; T4: one month after the third dose of COVID-19 vaccination; IQR, inter quartile range; eGFR, estimated glomerular filtration rate; CRP, C-reactive protein. eGFR was calculated by CKD-EPI index.

### 3.4 The immunosuppression therapy affected humoral response to the COVID-19 vaccine in KTR

We observed that 19/19 (100%) of KTR non-responders to the third dose were under mycophenolate mofetil as immunosuppressive therapy compared to 25/35 (71%) among responders (*p*=0.01). We could measure the pre-vaccination mycophenolate blood level in 38 patients and we found that, non-responders presented higher baseline blood levels of this immunosuppressive drug (4.6 [3.1-6.3] *vs.* 2.6 [0.8-3.1] *p*<0.001), and such levels correlated to a lower antibody production (r=-0.586, *p*<0.001) (**Supplementary Tables 2**, **3**; **Figure 2D**). We also explored whether the combination of the immunosuppressive drugs could be affecting the humoral response after three doses of vaccination, by categorizing the KTR population into three groups: I) receiving corticosteroids, tacrolimus and mycophenolate-mofetil (n=37; 68.5%); II) receiving corticosteroids, tacrolimus and mTOR inhibitors (n=6; 11.1%); III) receiving corticosteroids and tacrolimus (n=4; 7.4%). We observed a better response, regarding the seroconversion rates (100 *vs.* 56.7; *p*=0.042) in patients who were treated with mTOR inhibitors in triple combined therapy instead of the mycophenolate-mofetil. No differences were found between these triple combinations and the dual therapy of corticosteroids plus tacrolimus (**Supplementary Table 2**).

### 3.5 Inflammation, distribution of immune subsets and renal function also influenced the humoral response to COVID-19 vaccine in KTR

We obtained a data set comprised of the results from routine biochemical tests and hemograms which can provide insight into the immune response of patients to the vaccination. In addition to the demographical and immunosuppressive therapy related variables, we also analyzed inflammatory markers, kidney function markers and immune cell subsets. These variables are shown in **Table 2**, together with thymic function related parameters. The rest of the variables recorded from such routine tests can be found in the **Supplementary Table 3**. Patients that did not respond after three doses showed no differences in gender but tended to be older (*p*=0.068) and presented higher baseline levels of β2-microglobulin (*p*<0.001) (**Table 2**; **Figure 2E**). They showed also lower IgG levels (*p*=0.026) and lower counts of several immune cell populations, such as lymphocytes (*p*=0.012), B-cells (*p*=0.010), T-CD3 cells (*p*=0.025) and T CD4-cells (*p*=0.010) but higher frequency of neutrophils (*p*=0.001) (**Table 2**; **Figures 2F–J**). We also observed that seroconverted patients had higher eGFR and a lower proteinuria level (*p*=0.045 and *p*=0.025, respectively) (**Table 2**; **Figure 2K, L**). Correlations between all the continuous variables and the antibody titers after full vaccination are shown in **Supplementary Table 4**. Regarding sex, no differences were found in median T4 antibody titer in women compared to men (313 [152-2380] vs. 1647 [644-4480], *p*=0.210). Regarding correlations, we found negative correlations between the specific antibody titer at T4 and age (r=-0.407, *p*=0.002, serum level of creatinine (r=-0.314, *p*=0.022), proteinuria (r=-0.418, *p*=0.003), ferritin (r=-0.329, p=0.017), β2-microglobulin (r=-0.490, *p*<0.001), neutrophils% (r=-0.459, p=0.001) and mycophenolate-mofetil blood level (r=-0.586, p<0.001), whereas positive correlations were observed between the specific antibody titers at T4 and T3 (r=0.796, *p*<0.001), baseline eGFR (r=0.388, *p*=0.004), B-cells/µL (r=0.410, *p*=0.003) and sj/β TRECs ratio (r=0.399, *p*=0.017).

### 3.6 Predictive factors of humoral response to COVID-19 vaccine in KTR

We performed a multivariable analysis including, besides sex and age, variables related to each of the following categories: demographical parameters, inflammatory-related markers, thymic function-related factors, immune cell subsets, kidney function parameters and immunosuppression therapy. Taking into account that, due to the sample size of our study, we were limited to include up to eight variables in a multivariable analysis, we selected at least one representative variable from each mentioned category, according also to its biological relevance in the vaccination response context. The best fitted model showed that baseline creatinine serum level (βI coeff: -7214, *p*=0.011), β2-microglobulin (βI coeff: 2035, *p*=0.003) and T3 antibody titer (βI coeff: 20.5, *p*=0.036) were independently associated with T4 antibody titers, whereas CD4 T-cells tended to (βI coeff: -4.8, *p*=0.093) (**Table 3**). Furthermore, thymosin-α1 levels were independently associated with seroconversion after the full vaccination challenge in KTR (OR: 1.1, *p*=0.037) whereas the blood concentration of mycophenolate-mofetil tended to (OR: 0.3; *p*=0.060).

**Table 3:** Multivariable analysis of potential predictive factors (baseline determinations) in seroconversion and magnitude of humoral response to COVID-19 vaccine in KTR.

	T4 SARS-CoV-2 Trimeric-S IgG Levels (BAU/mL)			T4 Seroconversion	
	βI Coefficient (95% CI)	*p*-value		OR (95% CI)	*p*-value
Age (years)	-53.4 (-190, 83.8)	0.418	Age (years)	0.9 (0.7-1.1)	0.444
B-Cells (cells/mL)	7.6 (-20.5, 35.7)	0.573	Female (yes *vs.* no)	0.1 (0.01-10.6)	0.301
T-CD4 cells (Cells/mL)	-4.8 (-10.6, 0.9)	*0.093*	T-CD4 cells (Cells/mL)	1.0 (0.99-1.00)	0.972
Creatinine (mg/dL)	-7214 (-12495, -1934)	**0.011**	B-Cells (Cells/mL)	1.0 (0.9-1.0)	0.702
Mycophenolate-mofetil BL (mg/mL)	144 (-687, 975)	0.716	eGFR (mL/min)	0.9 (0.88-1.04)	0.336
Sj/β TRECs Ratio	26.3 (-233, 286)	0.831	Mycophenolate-mofetil BL (mg/mL)	0.3 (0.1-1.0)	*0.060*
β2-microglobulin (mg/mL)	2035 (806, 3463)	**0.003**	Tα1 (ng/mL)	1.1 (1.0-1.3)	**0.037**
T3 SARS-CoV-2 Trimeric-S IgG Levels (BAU/mL)	20.5 (1.6, 39.5)	**0.036**	Neutrophils (%)	0.9 (0.7-1.1)	0.327

Multiple linear regression (for the analysis of magnitude of response) and logistic (for seroconversion analysis) were applied. *p*-values <0.05 were considered statistically significant and shown in bold, whereas *p*-values between 0.1 and 0.05 were shown in italics. T4 one month after third dose of vaccine administration. T3 four months after the second dose and third dose administration. CI, Confidence interval; OR, Odds Ratio; BL, blood level; eGFR, glomerular filtration rate, was calculated by CKD-EPI index.

## 4 Discussion

In our cohort of kidney transplant recipients (KTR), the humoral response against the COVID-19 vaccine was seriously impaired, even though a third dose/booster significantly improved the seroconversion rate and antibody titers. Not surprisingly, immunosuppressive therapy played a critical role in both, the capability of responding and the humoral magnitude achieved. Remarkably, the capability of responding was associated with baseline thymic-related factors, such as the concentration of thymosin-α1, while the magnitude of the response was associated with different factors, including age and baseline renal and inflammatory status.

The KTR cohort showed an inferior response to the COVID-19 vaccine in comparison to the HC group, after two doses and even after an additional third dose, in the case of KTR. Although both groups were not age and sex matched, and were vaccinated with different vaccine mRNA platforms/doses, this result is consistent with previous data on similar cohorts (4, 19, 20). In fact, the seroconversion rate in KTR against the three-dose protocol COVID-19 vaccine is usually around 40% lower than that of healthy subjects and significantly lower than rates obtained for vaccines against other pathogens, such as influenza or pneumococcus (21, 22).

Due to the risk of developing severe disease in this population (23) and since the antibody titer achieved after COVID-19 vaccination has been related to a lower risk of infection and a better outcome of infection (24), new strategies are urgently needed to improve antibody titer after COVID-19 vaccination, and the vaccination efficiency in this population. In this line, in a recently published clinical trial (25), three different vaccination strategies have been tested in KTR without seroconversion after two or three doses of vaccination: a double dose of mRNA-1273, a heterologous vaccination (Ad26-COV2-S) and a transitory discontinuation of the mycophenolate mofetil administration as immunosuppressive therapy. Unfortunately, none of those strategies improved the vaccine response in comparison to the single dose of mRNA-1273, reinforcing the need for further research on potential modulators of vaccine response in this population. Moreover, in order to improve the protection against SARS-CoV-2 infection in KTR, the US and some European countries have approved the administration of SARS-CoV monoclonal antibodies to non-responder patients as a pre-exposure prophylaxis (26). So far, the advantage in KTR seems to be limited with the results differing according to the SARS-CoV-2 strain analyzed (27). Therefore, new approaches in this high-risk group of patients are still needed to improve the protection conferred by the vaccine administration.

In this study, we have analyzed immune-related factors potentially involved in the humoral response to vaccination. Interestingly, we found a strong positive correlation between the antibody titers after the two-doses schedule and the booster. Not surprisingly, we observed a poor response in those patients receiving mycophenolate-mofetil as immunosuppressive therapy. Moreover, age was related to lower antibody levels after three doses of vaccination. Both results are in accordance with those from a different KTR cohort receiving two doses of mRNA-1723 vaccine (26). Regarding the combined immunosuppressive therapy, we observed an improvement in the vaccination response when mTOR inhibitors were administered instead of mycophenolate-mofetil, similar to those data published in a KTR cohort receiving mycophenolate-mofetil in combination with tacrolimus (27). Currently, several trials intend to analyze the potential benefit of m-TOR inhibitors on the immune response. Our data suggest that such improvement could more probably reflect the negative effect of mycophenolate-mofetil than a beneficial effect of m-TOR inhibitors. However, the potential benefit of m-TOR inhibitors in the immune response cannot be discarded (28), since, as previously shown, mycophenolate substitution with mTOR inhibitor has a positive effect on virus clearance in kidney transplant recipients (29).

A better renal function and lower plasma levels of inflammation-related markers, such as neutrophils and B2-microglobulin, were associated with a better response in our KTR patients. B2-microglobulin is commonly elevated in KTR due to the immunosuppressive status and deteriorated kidney function (30). Furthermore, higher counts of immune cells, such as B-cells and T-CD4, as well as higher IgG levels, prior the immunization protocol favoured the response to the COVID-19 vaccine. The correct ratios of immune subsets are known to be critical during viral infections, such as the CD4/CD8 ratio in HIV-infection (7), or the total CD4+ and CD8+ T-cells producing IFN-γ or TNF-α as predictors of the immune response after vaccination against tuberculosis and herpes zoster (9).

In this line, we also explored the thymic function, which plays a main role in the maturation and functionality of T-cells and has been outlined in several contexts of limited immune responses, such as HIV-infection, as well as in aging (15, 31). As far as we know, the thymic function has not been yet explored in the context of the response to COVID-19 vaccine. We found a higher sj/β-TRECs ratio (which is the gold standard measure to asses thymic output) in seroconverted KTR after vaccination. Moreover, thymic function correlated with antibody titers in our group of patients. Globally, the KTR group showed a lower thymic output than the healthy group (data not shown). It is important to note that such difference could be easily explained by differences in sex and age between both groups. Nevertheless, thymic involution is suggested to be accelerated in kidney patients (32). Additionally, in our healthy group, no correlation was observed between the thymic output and the antibody titer after the COVID-19 vaccine (data not shown), but the limited availability of data from the thymic output in this group (n=11) precludes any conclusion about it. Furthermore, baseline thymosin-α1 (Tα1) plasma levels, an immunomodulatory hormone secreted by the thymus was positively associated with seroconversion. Tα1 has an immunologic-enhancing activity by the increase of CD4 and CD8 T-cell maturation and natural killer cell activation and is also considered as a humoral response enhancer (33). Furthermore, Tα1 has been related to a better recovery after SARS-CoV-2 infection and the efficacy of its administration for the treatment of COVID-19 is being explored (12, 13). This hormone has also been used in other infections, as in HIV-infection, with an immunomodulatory aim, and in HCV or pseudomonas infections with a therapeutic aim (34–36). Interestingly, Tα1 has also been used as an adjuvant in Influenza vaccine protocols (37, 38). Our results about the clinical benefit of Tα1 on COVID-19 vaccine response in KTR need to be confirmed in further studies. It would be important to evaluate the safety of Tα1 as a vaccination adjuvant in this clinical context since using immune stimulators in the context of transplanted patients could have negative consequences within the risk of organ rejection. However, Tα1 has been only administered to the solid organ transplant recipients for the treatment of serious complications such as cytomegalovirus infection and acute respiratory distress syndrome due to pneumonia (39, 40), showing a survival improvement without graft rejection.

We cannot conclude about the clinical protection achieved by the COVID-19 vaccine in our KTR cohort due to the low number of patients and the low rate of infection in our study period. However, from the five documented cases of SARS-CoV-2 infection after the administration of two doses of mRNA COVID-19 vaccine, three of them required hospital admission and one of those patients died due to a bilateral pneumonia. The other two patients presented mild symptoms. This is consistent with the rate of potentially seroprotected subjects in our cohort (50%), that is those patients reaching 260 BAU/mL, a threshold recently proposed for seroprotection (41), which is now being implemented by ours and others sanitary systems for clinical decisions.

We could not address the cellular response after vaccination in these patients, an important aspect to understand the full dynamics of the immune response after the immunization, and the role of thymic function in such response. Interestingly, an absence of correlation between SARS-COV-2 antibody titers and T cell response in kidney transplant recipients has been described (42).

Our study has additional limitations, such as a low sample size and a large number of variables analyzed which limits the statistical power to conclude about the immune predictors as well as about the role of the combinations of immunosuppressive drugs in the humoral response to COVID-19 vaccine in kidney transplant recipients. In addition, the thymic output data were obtained from PBMCs samples, rather than the isolated T-cell compartment, comprising thymic emigrants, which would therefore have increased the sensitivity of quantification. However, our novel findings could help in the design of future immunization strategies aiming the improvement of the COVID-19 vaccine response in these immunosuppressed patients and suggest the possibility of the use of thymosin-α1 as a vaccination adjuvant to improve their response following the next boosters in this risk population.

## Data availability statement

The raw data supporting the conclusions of this article will be made available by the authors, without undue reservation.

## Ethics statement

The studies involving human participants were reviewed and approved by Comité de Ética de la Investigación de los Hospitales Vírgen Macarena y Vírgen del Rocío de Sevilla. The patients/participants provided their written informed consent to participate in this study.

## Author contributions

Patient recruitment and clinical data collection (GB-B, AS-B, CG-C, MS-P, FG-R). Quantification of antibody titers (CL). Experiments of immune subsets (BS, IM-C, JL-R, MG-E). Experiments of biochemical and inflammatory markers (AA-R). Processing of samples and experiments of thymic function-related markers (AB-R, MP-B, IO-M, VG-R). Data base management and statistical analysis (AB-R, MP-B, YP). Manuscript preparation (AB-R, MP-B, YP). YP conceived the study, obtained funding and supervised the project. All authors contributed to the article and approved the submitted version.
