# Supplementary material for: Factors associated with the humoral response after three doses of COVID-19 vaccination in kidney transplant recipients
Source: Front Immunol. 2023 Feb 16;14:1099079. doi: 10.3389/fimmu.2023.1099079 (PMC9977967; doi:10.3389/fimmu.2023.1099079)
Supplement: Supplementary file 1 [file DataSheet_1.pdf]

SUPPLEMENTARY MATERIAL

FACTORS ASSOCIATED WITH THE HUMORAL RESPONSE AFTER THREE DOSES OF COVID-19 VACCINATION IN KIDNEY TRANSPLANT RECIPIENTS

Ángel Bulnes-Ramos, María del Mar Pozo-Balado, Israel Olivas-Martínez, Vanesa Garrido-Rodríguez, Gabriel Bernal-Blanco, Alejandro Suarez-Benjumea, Ana Isabel Álvarez-Ríos, Carmen Lozano, Carmen González-Corvillo, Marta Suñer-Poblet, Francisco Manuel González-Roncero, Berta Sánchez, Isabel Maldonado-Calzado, José Manuel Lara-Ruiz, María Francisca Gonzalez-Escribano, Yolanda María Pacheco.

SUPPLEMENTARY TABLES

Supplementary Table 1. Baseline demographic and clinical characteristics of kidney transplant recipients.

Variables	KTR n=74
Demographical parameters	
Age(years)	59 [47-66]
Sex (female)	23 (31.3)
Time from transplant (months)	59 [14-129]
Immunosuppression Therapy	
Corticosteroids	67 (90.5)
Mycophenolate	59 (79.7)
mTOR inhibitors	8 (10.8)
Tacrolimus	70 (94.6)
Cyclosporine	3 (4.1)
Thymoglobulin induction	7 (9.4)
Kidney Function Parameters	
Creatinine mg/dL	1.4 [1.1-1.9]
eGFR ml/min	50 [33-66]
Proteinuria mg/dL	246 [146-475]
Comorbidities	
High blood pressure	63 (85.1)
Diabetes	20 (27.0)
Cancer	13 (17.6)
Obesity	5 (6.8)

Categorical variables are expressed as n (%), and continuous variables as median [IQR]. IQR: interquartile range. mTOR: mammalian target of rapamycin; eGFR: estimated glomerular filtration rate.

Supplementary Table 2. Humoral response to COVID-19 vaccine in KTR according to the immunosuppressive therapy.

	T2 Response			T4 Response		
	Yes (n=27)	No (n=34)	p-value	yes (n=35)	no (n=19)	p-value
Immunosuppressive Therapy						
Thymoglobulin induction (yes)	0 (0)	27 (79.4)	0.022	2 (5.7)	4 (10.52)	*0.087*
Corticosteroids (yes)	24 (88.9)	30 (88.2)	0.937	33 (94.3)	16 (84.2)	0.223
Mycophenolate (yes)	19 (70.4)	28 (82.4)	0.296	25 (71.4)	19 (100)	**0.010**
Mycophenolate blood level (ng/mL)*	3.1 [1.2-3.9]	3.1 [2.2-5.1]	0.822	2.6 [0.8-3.1]	4.6 [3.1-6.3]	**<0.001**
Cyclosporine (yes)	1 (3.8)	1 (3.2)	0.899	2 (6.5)	0 (0)	0.271
Tacrolimus (yes)	26 (96.3)	32 (94.1)	0.696	33 (94.3)	19 (100)	0.288
Tacrolimus blood level (ng/mL)	7.2 [5.2-9.3]	7.8 [6.3-9.0]	0.654	6.8 [5.2-8.8]	7.2 [6.5-8.6]	0.497
mTOR Inhibitors (yes)	4 (16.0)	3 (9.7)	0.477	5 (16.7)	0 (0)	*0.067*
Combined Immunosuppressive Therapy						
Cort+Tacro+Mycophenolate	15 (40.5)	22 (59.5)	0.467	21 (56.8)	16 (43.2)	*0.067*
Cort+Tacro+mTORi	4 (66.7)	2 (33.3)	0.245	5 (100)	0 (0)	*0.084*
Cort+Tacro	4 (57.1)	3 (42.9)	0.466	4 (100)	0 (0)	0.126
Others	4 (40)	6 (60)	0.767	4 (57.1)	3 (42.9)	0.767

Categorical variables are expressed as n (%), and continuous variables as median [IQR]. Parameters were compared using Mann-Whitney *U*-test or χ^2 test when proceeded. *p*-value <0.05 were considered statistically significant and shown in bold, whereas *p*-values between 0.1 and 0.05 were shown in italics. T2: One month after second dose of COVID-19 vaccine; T4: one month after the third dose of COVID-19 vaccine; IQR: inter quartile range. mTOR: mammalian target of rapamycin, Cort: corticoids, Tacro: Tacrolimus, mTORi: inhibitors of mTOR. *N= 38

Supplementary Table 3. Analysis of soluble markers in KTR depending of their humoral response to COVID-19 vaccination.

	T2 Response			T4 Response		
	Yes (n=27)	No (n=34)	*p*-value	yes (n=35)	no (n=19)	*p*-value
Nutritional status and lipid metabolism						
Albumin, g/dL	4.7 [4.3-4.9]	4.8 [4.5-5.2]	0.212	4.7 [4.3-5.2]	4.8 [4.6-4.9]	0.275
Cholesterol, mg/dL	202 [159-215]	181 [156-194]	0.177	181 [147-207]	183 [156-207]	0.693
HDL cholesterol, mg/dL	61.5 [46.0-73.0]	59 [49-69]	0.879	54.5 [43.7-72.0]	67.0 [47.0-76.0]	0.229
LDL cholesterol, mg/dL	108 [81-130]	94 [74-107]	0.192	92 [68-111]	87 [67-112]	0.923
Triglycerides, mg/dL	137 [114-169]	116 [90-153]	0.153	134 [113-172]	122 [93-162]	0.519
B12 Vitamin, pg/mL	450 [360-651]	396 [301-534]	0.225	410 [307-643]	516 [413-592]	0.281
Folic Acid, ng/mL	6.1 [3.7-7.5]	6.3 [4.6-9.2]	0.271	6.3 [4.4-8.3]	6.4 [4.8-9.9]	0.610
Transferrin, mg/dL	268 [246-300]	245 [212-302]	0.915	245 [214-290]	250 [230-299]	0.604
Soluble transferrin receptor, mg/L	4.0 [3.4-5.4]	4.3 [3.3-4.9]	0.988	4.3 [3.5-5.0]	4.0 [2.9-5.0]	0.545
Transferrin saturation index, %	24 [17-32]	22 [19-34]	0.915	23 [19-35]	24 [18-35]	0.923
Immunoglobulins						
IgA, mg/dL	158 [132-378]	204 [149-268]	0.897	224 [151-319]	154 [121-237]	*0.051*
IgM, mg/dL	105 [71-157]	98 [63-148]	0.766	105 [77-155]	75 [59-115]	*0.065*
IgG, mg/dL	1086 [863-1196]	962 [772-1109]	0.275	1038 [885-1232]	758 [714-1074]	**0.026**
Micronutrients						
Calcium, mg/dL	10.1 [9.7-10.3]	10.2 [9.8-10.6]	0.254	10.2 [9.7-10.5]	10.1 [9.7-10.5]	0.923
Phosphorus, mg/dL	3.1 [2.7-4.1]	3.7 [3.2-4.1]	*0.049*	3.2 [2.6-3.7]	3.9 [3.2-4.2]	**0.032**
Sodium, mEq/L	146 [144-149]	147 [144-150]	0.515	147 [144-149]	145 [142-149]	0.406
Potassium, mEq/mL	4.7 [4.2-4.9]	4.7 [4.4-5.2]	0.363	4.6 [4.1-5.0]	4.8 [4.4-5.2]	0.133
Magnesium, mg/dL	1.9 [1.7-1.9]	1.8 [1.6-1.9]	0.789	1.7 [1.6-1.9]	1.9 [1.7-2.0]	**0.042**
Iron, µg/dL	87 [61-112]	76 [59-105]	0.454	75 [60-99]	79 [58-114]	0.855

Continuous variables are expressed as median [IQR]. Comparisons were tested by using Mann-Whitney or *U*-test when proceeded. *p*-value <0.05 were considered statistically significant and shown in bold, whereas *p*-values between 0.1 and 0.05 were shown in italics. T2: one month after second dose of COVID-19 vaccination; T4: one month after the third dose of COVID-19 vaccination; IQR, inter quartile range.

Supplementary Table 4. Associations between the specific antibody titer after the third dose of vaccine and all the studied parameters measured before vaccination in KTR patients.

T4 SARS-CoV-2 Trimeric-S IgG Levels (BAU/mL) (n=54)					
**Variable**	**r-coefficient**	***p*-value**	**Variable**	**r-coefficient**	***p*-value**
Demographical parameters			Soluble Markers (cont.)		
Age, years	-0.407	**0.002**	Soluble transferrin receptor, mg/L	-0.011	0.938
Time post transplantation, months	-0.010	0.948	Transferrin saturation index, %	-0.022	0.875
Kidney Function Markers			Beta-2-microglobuline, mg/L	-0.490	**<0.001**
Creatinine, mg/dL	-0.314	**0.022**	CRP, mg/L	-0.045	0.752
eGFR, mL/min	0.388	**0.004**	Thymic function		
Proteinuria, mg/dL	-0.418	**0.003**	Tα1, ng/mL	0.180	0.205
Soluble Markers			sj/β-TRECs RATIO	0.399	**0.017**
T3 SARS-CoV-2 Trimeric-S IgG levels- BAU/ml	0.796	**<0.001**	Telomere Relative Length	-0.084	0.560
IgA, mg/dL	0.139	0.326	Immunosuppression Therapy		
IgM, mg/dL	0.176	0.212	Mycophenolate BL, ng/mL*	-0.586	**<0.001**
IgG, mg/dL	0.326	**0.018**	Tacrolimus BL, ng/mL$^{\neq}$	0.215	0.363
Albumin, g/dL	-0.244	*0.082*	Immune cells populations		
Cholesterol, mg/dL	0.072	0.611	Neutrophils, cells/µL	-0.193	0.199

HDL cholesterol, mg/dL	-0.157	0.266	Neutrophils, %	-0.459	**0.001**
LDL cholesterol, mg/dL	0.114	0.423	Lymphocytes, cells/µL	0.273	*0.052*
Triglycerides, mg/dL	0.176	0.213	B-cells, cells/µL	0.410	**0.003**
Homocysteine, mg/dL	-0.255	*0.068*	B-cells, %	0.215	0.130
B12 Vitamin, pg/mL	-0.110	0.439	T CD3, cell/µL	0.239	*0.091*
Folic Acid, ng/mL	-0.007	0.960	T CD3, %	-0.013	0.930
Calcium, mg/dL	-0.085	0.551	T CD4-cells, cells/µL	0.263	0.062
Phosphorus, mg/dL	-0.430	**0.001**	T CD4, %	0.120	0.400
Sodium, mEq/mL	0.044	0.759	T CD8-cells, cells/µL	0.176	0.216
Potassium, mEq/mL	-0.271	*0.052*	T CD8, %	-0.009	0.952
Magnesium, mg/dL	-0.249	*0.075*	NK-cells, cells/µL	0.057	0.692
Iron µg/dL	0.081	0.568	NK cells, %	-0.084	0.556
Ferritin, ng/mL	-0.329	**0.017**	CD4/CD8 ratio, median	0.090	0.530
Transferrin, mg/dL	0.171	0.324			

Spearman correlations were teste and Spearman's correlation coefficient (r) and exact *p*-value are given. *p*-value <0.05 were considered statistically significant and shown in bold, whereas *p*-values between 0.1 and 0.05 were shown in italics. BL, blood level; eGFR: glomerular filtration rate, calculated by CKD-EPI index; T4: one month after the third dose of COVID-19 vaccine. *N= 38, $^{\neq}$N=45

SUPPLEMENTARY FIGURE

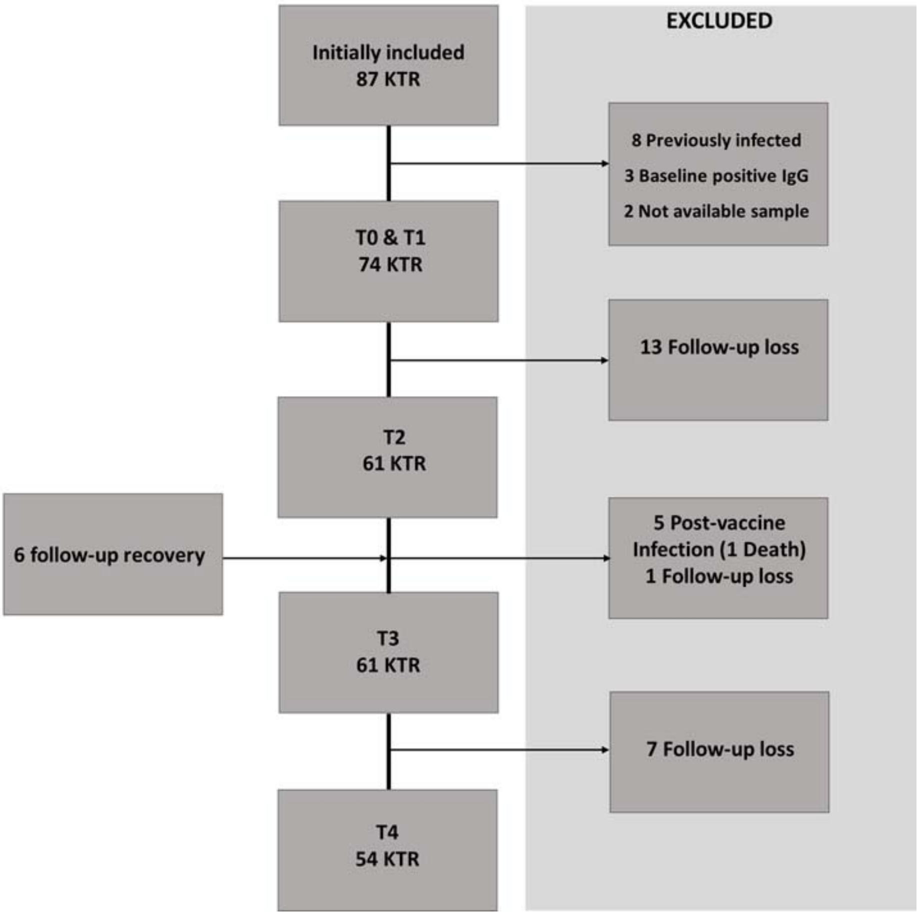

Supplementary Figure 1. **Flow-chart of study.**

Eighty-seven KTR were recruited before vaccination, however, eight of them were excluded by previous SARS-CoV-2 infection (confirmed by PCR) and three more for positive Trimeric-S IgG (≥ 33.8 BAU/mL) at baseline. Hence, seventy-four were finally included in the analyses at baseline and 3-4 weeks after the first dose (T0 and T1). We lost the follow-up of 13 patients one month after the second dose (T2). After this time-point, five patients had diagnosed SARS-CoV-2 infection (confirmed by PCR), one of them died due to a bilateral pneumonia due to the infection. In addition, one KTR lost the follow-up whereas six patients were recovered before T3. One month after receiving the third dose (T4), seven more patients were lost to the follow-up.
